# A new look at osteoarthritis: Threshold potentials and an analogy to hypocalcemia

**DOI:** 10.3389/fragi.2023.977426

**Published:** 2023-03-08

**Authors:** P. Van Gelder, E. Audenaert, P. Calders, L. Leybaert

**Affiliations:** ^1^ Department of Rehabilitation Sciences, Ghent University, Ghent, Belgium; ^2^ Department of Orthopaedic Surgery and Traumatology, Ghent University, Ghent, Belgium; ^3^ Department of Basic and Applied Medical Sciences (BAMS), Physiology Group, Ghent University, Ghent, Belgium

**Keywords:** osteoarthiritis, membrane proteins, potentials, voltage sensors, cartilage

## Abstract

Cartilage is a tissue that consist of very few cells embedded in a highly negatively charged extracellular matrix (ECM). This tissue is dealing with several electrical potentials which have been shown to control the production of ECM. Cartilage is present at joints and is constantly prone to degradation. Failing to repair the damage will result in the occurrence of osteoarthritis (OA). This perspective aims to link biophysical insights with biomolecular research in order to provide an alternative view on the possible causes of OA. Firstly, we hypothesize the existence of a threshold potential, which should be reached in order to initiate repair but if not met, unrepaired damage will evolve to OA. Measurements of the magnitude of this threshold electrical potential would be a helpful diagnostic tool. Secondly, since electrical potential alterations can induce chondrocytes to synthesize ECM, a cellular sensor must be present. We here propose an analogy to the hypocalcemia ‘unshielding’ situation to comprehend electrical potential generation and explore possible sensing mechanisms translating the electrical message into cellular responses. A better understanding of the cellular voltage sensors and down-stream signalling mechanisms may lead to the development of novel treatments for cartilage regeneration.

## Introduction: Cartilage tissue, the negatively charged sponge

**FIGURE 1:**
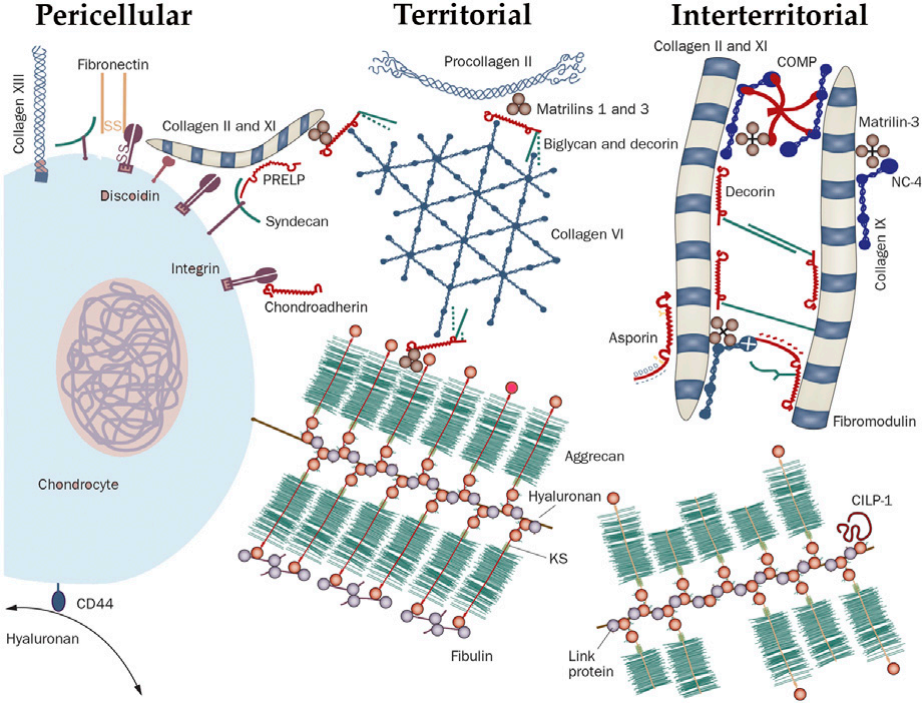
The molecular organization of cartilage around a chondrocyte. The cartilage matrix is arranged into different zones defined by their distance from the cell. The space immediately around the chondrocyte, that is the zone where molecules interact with cell surface receptors, is called the pericellular matrix (PM); for example, hyaluronan binds the receptor CD44. Next to the PM lies the territorial matrix and at largest distance from the cell is the interterritorial matrix whereby each zone is defined by the types of collagens and the collagen-binding proteins. Abbreviations: CILP, cartilage intermediate layer protein 1; COMP, cartilage oligomeric matrix protein; CS, chondroitin sulfate; KS, keratan sulfate; PRELP, proline-arginine-rich end leucine-rich repeat protein; hyaluronan (HA); CHAD, Chondroadherin; HS-PG, heparan sulphate proteoglycan; MAT, matrilin; NC4, non-collagenous domain 4. Reproduced from Heinegård. (2009) with permission from Wiley & Sons.

Cartilage is a flexible connective tissue that can be found throughout the animal kingdom in many places of the body where it serves as a shock absorber. Compared to other connective tissues, cartilage has a very slow turnover and extremely slow cell growth and is not easy to repair. Another remarkable feature of cartilage is the stunningly low cell content of <2.5% (Hunziker et al., 2002; Quinn et al., 2005; Quinn et al., 2013) of which both chondrocytes and chondroblasts (the immature cells which can differentiate into chondrocytes), are involved in the maintenance and repair of the extracellular matrix (ECM) of the cartilage. This ECM is composed of proteoglycan and elastic fibers such as different types of collagen and is divided in different zones around the cells (Figure 1; Heinegård, 2009). The proteoglycan is a chain of glycosaminoglycans (GAG) which are covalently attached to various core proteins such as the family of large extracellular chondroitin sulfate proteoglycans like aggrecan or the small leucine-rich core protein family such as decorin, biglycan, fibromodulin, lumican and epiphycan or the basement membrane proteoglycan, perlecan (Knudson and Knudson, 2001). This unit is than linked to hyaluronan *via* a link protein. Hyaluronic acid and other sulphated GAG (e.g., chondroitin sulphate) contains negative charges that attract high charge density countercharges such as Ca^2+^ and Mg^2+^. These negative charges are the so called fixed negative charges (FNC) as opposed to the mobile positive charges of ions dissolved in the extracellular fluid phase (Athanasiou et al., 2017). Under physiological loading conditions, chondrocytes maintain the balance between degradation and synthesis of matrix components. When loading becomes excessive or in case of injury, synthesis will lag in comparison with degradation, causing joint degradation and eventually osteoarthritis (Chen et al., 2013).

## A heavily charged ECM: The case for potentials in cartilage

Cartilage is an elastic material with a dynamic elastic modulus of ∼2.6 MPa as measured by indentation-type atomic force microscopy on porcine samples, using a micrometer sized tip. By contrast, nanometer sized tips used to explore elasticity at the fine structure level report 100 fold lower values (Stolz et al., 2004). The origin of these mechanical parameters was described in what is known as the “triphasic theory,” through changes in properties in response to deformation and occurrence of stress fields in a biphasic fluid-solid state (the interstitial fluid and the ECM) with a tertiary phase of mobile ions (Na^+^ and Cl^−^ in most models) under mechanical load. This theory indicated that the Donnan osmotic pressure, the chemical-expansion stress (originating from the repulsive forces of the compressed negative GAG molecules) and the solid matrix elastic stress are the main determinants of overall compression stiffness of cartilage (Lai et al., 1991; Lai et al., 2000; Wan et al., 2004). In another approach, Bushmann and Grodzinsky demonstrated that the pressure build-up in cartilage is mainly due to chemical-expansion stress and is best described by a molecular level Poisson Boltzmann model instead of a macroscopic Donnan model (Buschmann and Grodzinsky, 1995). To complicate things, a positive electrostatic field has been reported to be created outside the cartilage matrix by convection-induced positive charge accumulation at the synovial side of the cartilage-synovial fluid interface (Quenneville and Buschmann, 2005); data on such convection-induced polarization are however limited. By contrast, *in vitro* experiments on cartilage exposed to mechanical force clearly show the appearance of a well-documented “streaming potential” that can be measured by an extracellular electrode placed in the center of a cartilage disc preparation, relative to a bath electrode positioned at the outer edge (Kim et al., 1995). *Ex vivo* experiments on isolated porcine cartilage demonstrated the appearance of streaming potentials of up to −5.5 mV (Schmidt-Rohlfing et al., 2002), which correlated with the amount of compression pressure applied, with higher pressures resulting in larger potentials. Similar potentials (−5.3–−6.9 mV) were measured in human lumbar spine discs and were found to be anisotropic in nature and to reflect the degree of cartilage degeneration with larger streaming potential magnitudes being found in less degenerated discs (Gu et al., 1999); these results were confirmed in bovine experiments (Fujisaki et al., 2011). Moreover, the magnitude of the streaming potentials decreased after successive loading cycles measured in human volunteers using electroarthrography (Zhu et al., 2016). Interestingly, these experiments demonstrate that the streaming potential is present in different, articular and fibrous, cartilage types and that its magnitude decreases with degeneration of the cartilage.

## The fascinating effect of electrical potentials on cartilage

Evidence has become available demonstrating that electrical fields may influence chondrocyte function and cartilage properties. In a compression study on bovine cartilage explants it was shown that the synthesis of aggrecan was increased in the regions with the highest interstitial fluid flow (Buschmann et al., 1999). No electrical parameters were recorded in this study, but follow-up work by others later demonstrated that capacitatively coupled electrical signals were able to upregulate chondrocyte matrix genes and products such as Type II collagen and aggrecan in bovine articular chondrocytes (Wang et al., 2004), which was further corroborated in a 2006 study, also on bovine cartilage, where an increase in mRNA for collagen and proteoglycans was observed in response to externally applied electrical fields (Brighton et al., 2006). The team of Alan Grodzinsky had already in 1995 demonstrated an increased proteoglycan production under influence of streaming potentials in bovine explants. The application of 10 mA/cm^2^ with a 100–1,000 Hz frequency resulted in significantly increased protein synthesis as measured by an increased incorporation of ^35^S–methionine in articular cartilage explants (MacGinitie et al., 1994). Interestingly, electrical currents had a positive effect on the proliferation of chondrocytes but not on mature hypertonic chondrocytes which are located deeper in the cartilage matrix where fluid currents and shear potentials are less pronounced compared to the cartilage surface (Nogami et al., 1982). Two studies by the team of Maretto Esquisatto demonstrated that a clear and remarkable recovery of artificially induced (by a punch) damaged rat cartilage was observed after 35 days of electrical stimulation, resulting in significantly increased proteoglycan content as compared to non-treated samples (de Campos Ciccone et al., 2013; Zuzzi et al., 2013). Other studies demonstrated enhanced expression levels of type II collagen, aggrecan, proteoglycan and Sox9, driving oscillations in intracellular Ca^2+^ and ATP concentration (Fitzgerald et al., 2004), and significantly increasing TGF-β1 and BMP2 (Brighton et al., 2006; Kwon et al., 2016), which may be ultimately involved in the repair mechanisms of the cartilage.

## Making sense of the streaming potential: The whodunit of membrane proteins

The obvious question that follows is how chondrocytes exactly sense the mechanical loading induced electrical signals. Currently, there is indeed a gap between the macroscopic events leading to the appearance of electrical potentials in cartilage and the knowledge of membrane protein channels/sensors on the microscopic level that might be activated by these potentials. Chondrocytes express a substantial array of ion channels, including voltage-gated Na^+^, K^+^ and Ca^2+^ channels (Mobasheri et al., 2012; Mobasheri et al., 2019). However, the resting membrane potential (Vm) of these cells is rather depolarized, ranging from −40 to −10 mV as measured in different organisms (Sugimoto et al., 1996; Clark et al., 2010; Funabashi et al., 2010; Lewis et al., 2011). At such depolarized potentials, voltage-gated Na^+^ and Ca^2+^ channels may become inactivated and therefore reside in a closed state (e.g., chondrocytic Cav3.2 channels start to inactivate from −80 mV on; Monteil et al., 2015). Interestingly, voltage sensing is not a prerogative of ion channels as G-protein coupled receptors (GPCRs) may also express voltage sensors (Bezanilla, 2008). E.g., muscarinic receptors display voltage-dependent responses, with depolarizing stimuli enhancing M1-type receptor responses while reducing those of M2 receptors (Ben-Chaim et al., 2006); in chondrocytes, this may lead to increased GAG synthesis (Lind et al., 1998). The ubiquitous P2Y_12_ purinergic receptor, present in chondrocytes and involved in their migration (Szustak and Gendaszewska-Darmach, 2020) is another example where voltage sensing through GPCRs may impact function (Zhang et al., 2020).

Next to voltage sensing membrane proteins, changes in Vm may have a more direct impact by affecting the driving force for transmembrane transport of electrically charged substances. In fact, any membrane transporter or channel that carries net charge over the plasma membrane, called “electrogenic transport,” will be affected by changes in Vm or by local charge accumulation. This includes transporters like the Na^+^/K^+^-ATPase, the plasma membrane Ca^2+^-ATPase and the Na^2+^/Ca^2+^ exchanger, as well as NMDA receptor channels, TRP channels and Ca^2+^ channels present in chondrocytes (Mobasheri et al., 2019). As such, currents through, e.g., TRPV5 channels may be altered by streaming potentials and thereby affect chondrocyte volume regulation (Lewis et al., 2011). Also TRPV4 channels are involved in chondrocyte Ca^2+^ signaling and mechanosensing (Agarwal et al., 2021), together with piezo-1 and piezo-2 mechanosensitive channels (Du et al., 2020). The Ca^2+^-sensing receptor is another well characterized player in bone but also cartilage development and remodeling (reviewed in Goltzman and Hendy, 2015). This G-protein coupled receptor (GPCR) is activated by extracellular Ca^2+^ elevation and translates to intracellular IP_3_/Ca^2+^ signaling. Activation of the Ca^2+^-sensing receptor is involved in the pathophysiology of osteoarthritis (Burton et al., 2005); however, in the context of ‘Ca^2+^-unshielding’ discussed below, this receptor would become less activated and thus less likely to be involved in the sensing of streaming potentials.

## Hypocalcemia: A particular case of charge shielding and sensors

**FIGURE 2:**
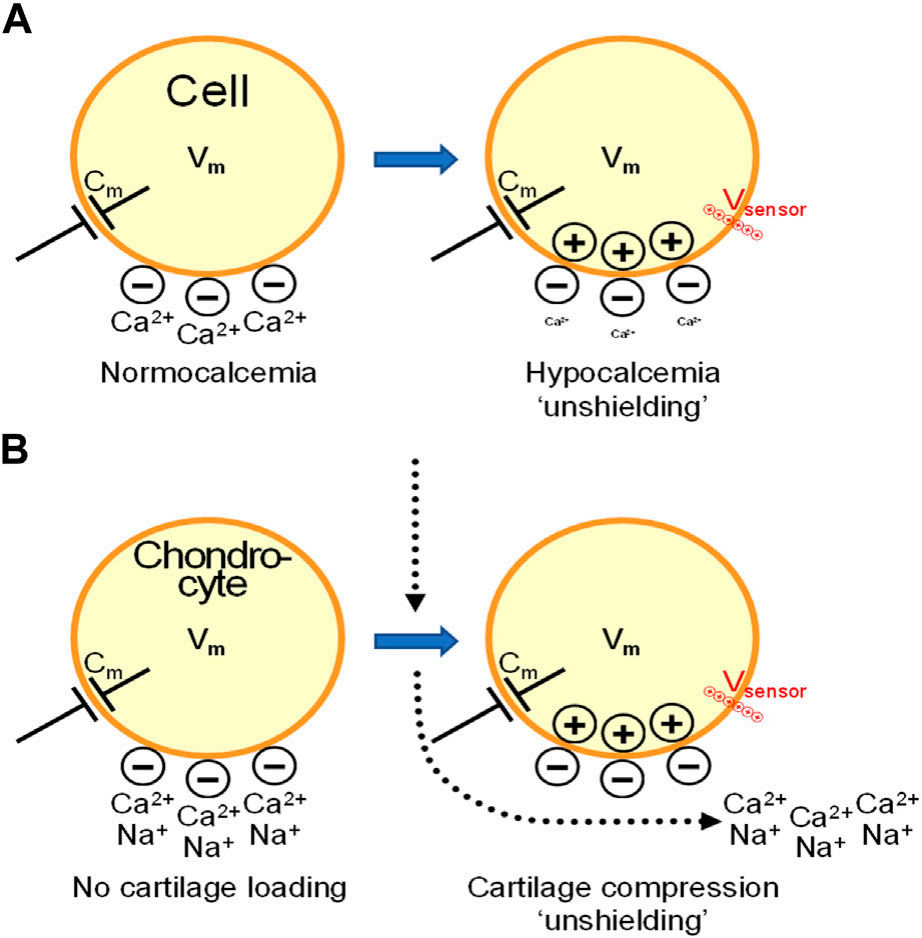
Cartilage compression may generate intracellular accumulation of positive charges by mechanisms that resemble the “unshielding effect” in hypocalcemia. From streaming potentials to functional alterations in chondrocytes and cartilage degeneration. **(A)**. In hypocalcemia, the decreased extracellular Ca^2+^ concentration exposes fixed negative charges in the glycocalyx surrounding the cells, effectively unshielding the outside negativity. Due to membrane capacitance (C_m_), the uncovered negativity attracts positive charges inside the cell. Voltage sensors of plasma membrane ion channels feel this local positive charge accumulation inside and react in very much the same way as if a depolarizing Vm stimulus would be applied. In muscle and nerves, this causes hyperexcitability because the set-point of voltage sensitive Na^+^ channels is shifted closer to the activation threshold upon hypocalcemia. **(B)**. In cartilage, compression triggers interstitial fluid flow containing anions, e.g., Cl^−^, bicarbonate, and cations, mainly Na^+^ and Ca^2+^. At the center of pressure loading (vertical dotted arrow), this creates a centrifugal movement of Na^+^ and Ca^2+^ from the center to peripheral zones (bended arrow) thereby leaving behind fixed negative charges that become unshielded and lead to a local negative streaming potential. Inside the chondrocytes, this creates positive charge accumulation that, as in hypocalcemia, brings voltage sensors closer to their activation threshold. This may affect voltage sensitive ion channels but also GPCRs and electrogenic transports, leading to functional consequences as a result of altered signaling and activation of gene programs. In OA, streaming potentials decrease in magnitude and we hypothesize that the threshold for activating repair signaling is not reached, leading to progressive cartilage degeneration.

Of note, it is important to distinguish local effects of mechano-loading induced streaming potentials from Vm changes. Therefore we like to present an educated guess on how macroscopic induced changes in cartilage might affect membrane proteins by comparing the situation with a very well known paradigm of altered charge accumulation in the condition of hypocalcemia. Interestingly, the fixed ECM-linked negative charge accumulation associated with cartilage loading will influence membrane voltage sensors in very much the same way as occurs in hypocalcemia (Figure 2). In hypocalcemia, Ca^2+^ occupation of fixed negative charges in the glycocalyx decreases, effectively unshielding the outside negativity, which is reminiscent of the situation in cartilage during mechanical compression where mobile positive charges, in particular Ca^2+^ are expulsed and the negative charges remain unshielded outside the chondrocyte plasma membrane. Due to the membrane capacitance, the *de novo* appearing outside negativity attracts positive charges inside. Membrane voltage sensors may sense the positive charge accumulation inside and react in very much the same way as if an intracellular depolarizing Vm stimulus would be applied. In muscle, this causes hyperexcitability because voltage sensitive Na^+^ channels come closer to their activation threshold in hypocalcemia. Likewise, in mechano-loaded chondrocytes, local electrostatic effects that lead to streaming potentials may affect voltage sensors of ion channels and GPCRs, and alter the driving force and activity of electrogenic transports. Moreover, exogenously applied electrical fields may superimpose on these local charge effects thereby reaching a threshold for activating cartilage recovery programs. Of the above mentioned chondrocyte channel family, voltage-gated Ca^2+^ channels stand out in their ability to induce ECM gene transcription and increased production of ECM components (Xu et al., 2009). These authors observed that electrical stimulation resulted in a 3-4 fold up-regulation of aggrecan and type II collagen mRNA in chondrocytes, which was abolished by voltage-gated Ca^2+^ channel blockers. Weber and Waldman, (2014) furthermore found a positive correlation between Ca^2+^ signaling and matrix synthesis under mechanical loading conditions. Chicken chondrocytes express several types of voltage-gated calcium channels, including L-type (Ca_v_1.2 and Ca_v_1.3), R-type (Ca_v_2.3) and T-type (Ca_v_3.1, Ca_v_3.2 and Ca_v_3.3). Most interestingly, L-type Ca^2+^ channel inhibition with nifedipine was reported to completely abrogate chondrogenesis (Fodor et al., 2013). These data suggest that L-type Ca^2+^ channels are functional and not completely inactivated in chondrocytes as suggested by their depolarized Vm referred to higher. Thus, Ca^2+^ channels may play a role in acute or delayed (gene expression-dependent) chondrogenetic responses, pressing for further studies. Here, we propose that the streaming potential and consequent intracellular signaling, including Ca^2+^ signaling, may tilt the balance towards chondrogenesis and repair.

## Cartilage in a see/saw mode, a cliffhanger towards osteoarthrosis?

It is known that the magnitude of the streaming potentials decreases with the degree of cartilage degradation (Garon et al., 2002; Légaré et al., 2002). Under normal healthy conditions, the streaming potential is apparently able to stimulate the biogenesis of cartilage and counter the degradation by wear and tear in a continuous cycle. One of the current recommendations for people at risk of developing OA is exercise (Roos and Dahlberg, 2005; Racunica et al., 2007; Ravalli et al., 2019; Kong et al., 2022). Exercise may exert this effect by enhancing the magnitude of streaming potentials thereby stimulating chondrocytes to produce new ECM. This raises the question whether there exists a threshold streaming potential which, if not reached, is unable to stimulate cartilage repair. The damage inflicted to the cartilage can result in a decreased number of active chondrocytes, which could explain a diminished repair potential of the damaged matrix. However, microscopic studies performed by Häuselmann and his team (Hunziker et al., 2002; Quinn et al., 2005; Quinn et al., 2013) revealed an extraordinary low cell volume density of only between 1% and 2.5% in articular cartilage, which indicates that most of the damage will be inflicted on the matrix structure thereby decreasing the magnitude of the streaming potential. Interestingly, cell volume measurements in healthy and damaged cartilage did not find a decreased number of cells but rather a decreased volume regulation which resulted in an increased cell volume percentage (Bush and Hall, 2003). Therefore we hypothesize that once the cartilage has reached a critical degree of damage and loss of negatively charged ECM, the potential may dip below a putative threshold and thereby mark the point of no return where the pathology further develops into a full blown OA condition.

## Conclusion: OA and streaming potentials, a bucket list

In the wake of several papers reporting on streaming potentials, researchers have developed devices to measure this potential and to correlate this to a progressive degradation of the cartilage. The teams of Grodzinsky and recently Buschmann have developed a quantitative electrical impedance analysis method for cartilage degradation by measuring the electrolyte content in several species (canine, human, bovine; US patent 6735468B2; Frank et al., 2011; Changoor et al., 2020). The sensitivity and reliability of such approaches is still limited with substantial variability of the measured potentials. Novel technologies such as nano-device carbon electrodes may however yield more stable less-invasive approaches, allowing continuous monitoring for patients at risk of developing OA (e.g., obese patients). Thus at the engineering front there is definitely a need for standardization and more precise measurements. In terms of theoretical insights there are still many questions open related to the generation of streaming potentials, the contribution of convection-induced polarization effects at the cartilage-synovial fluid interface, and the impact of these complex effects on cellular signaling. Will it be possible to produce a better model that can cope with this complexity and that is backed up by experimental observations, e.g., by measurements on chondrocytes embedded in artificial polymers with an adjustable and known density of fixed negative charges? Can the hypothetical threshold potential proposed here be documented *in vivo*? Can cartilage biogenesis be stimulated by applying external electrical fields, thereby mimicking the effects of streaming potentials ? Is it possible to overcome the hypothetical threshold potential by an external electrical field ? Can we apply external electrical stimulation combined with rehabilitation to counter the progressive cartilage degradation of patients that have subthreshold potentials ? Alternatively, can we trick the chondrocytes by pharmacological intervention to stimulate the production of ECM by targeting the membrane channel or transport proteins ? Most importantly, to trick the chondrocytes one has to identify the sensor. Here we are favouring a calcium channel as sensor given the effect of Ca2+ on ECM production but this does not exclude that other channel types may be sensitive to the streaming potential. Further research in this exciting field may certainly stimulate new findings and avenues for therapeutic interventions in OA patients.

## Data Availability

The original contributions presented in the study are included in the article/Supplementary Material, further inquiries can be directed to the corresponding author.
